# Data augmentation in a triple transformer loop retrosynthesis model

**DOI:** 10.1039/d5dd00465a

**Published:** 2026-01-21

**Authors:** Yves Grandjean, David Kreutter, Jean-Louis Reymond

**Affiliations:** a Department of Chemistry, Biochemistry and Pharmaceutical Sciences, University of Bern Freiestrasse 3 3012 Bern Switzerland jean-louis.reymond@unibe.ch

## Abstract

Reactions in the US Patent Office (USPTO) are biased towards a few over-represented reaction types, which potentially limits their usefulness for computer-assisted synthesis planning (CASP). To obtain an equilibrated dataset, we applied retrosynthesis templates to USPTO molecules as products (P) to generate starting materials (SM). We then used transformer T2 from our recently reported triple transformer loop (TTL) retrosynthesis model to predict reagents (R) for the SM → P reaction. Finally, we validated the prediction by requesting a high confidence prediction (>95%) for the prediction of P from SM + R by TTL transformer T3. We generated up to 5000 reactions per template, resulting in 27.5m validated fictive reactions covering the chemical space of the original USPTO dataset. To exemplify the use of this dataset, we demonstrate that a single-step retrosynthesis transformer model trained on a template equilibrated subset of 1 097 374 fictive reactions outperforms the corresponding model trained on USPTO reactions only.

## Abstract 2

To mitigate bias in the USPTO dataset, we generated fictive reactions from USPTO templates and validated them with a triple transformer loop. Retrosynthesis models trained on this data outperform those trained on USPTO alone.

## Graphical abstract

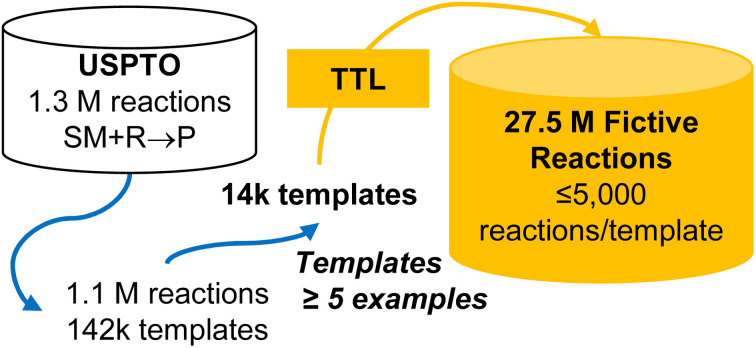

## Introduction

The challenge of computer-assisted synthesis planning (CASP) consists of training neural networks and related models with data on organic reactions to automatically propose possible retrosyntheses of any molecule of interest.^1–21^ One of the key current limitations in this field is the dataset of reactions available for training, which is based on data extracted from the publicly available US Patent Office (USPTO).^22^ For instance, classifying reactions in this dataset by reaction templates, as discussed below, reveals a typical power-law distribution in which the majority of USPTO reactions correspond to a small number of templates, while many templates only possess very few examples. This imbalance reflects a bias towards reaction types frequently used in the patent literature, as well as the fact that some reaction types are simply rare and only documented sparsely, including in the primary literature.

This relative sparsity of reaction data has been addressed by data augmentation using SMILES randomization^23^ and more directly by applying reaction templates (abstracted transformation rules encoded in SMARTS or SMIRKS) to molecules from various sources to generate fictive reactions that are then added to the training data to augment CASP tool performance.^24–26^ The decision to apply a reaction template to a molecule and/or to accept or reject the generated fictive reaction relies on molecular similarity between the molecule or generated reaction and the database examples from which the template was originally extracted.

**Fig. 1:**
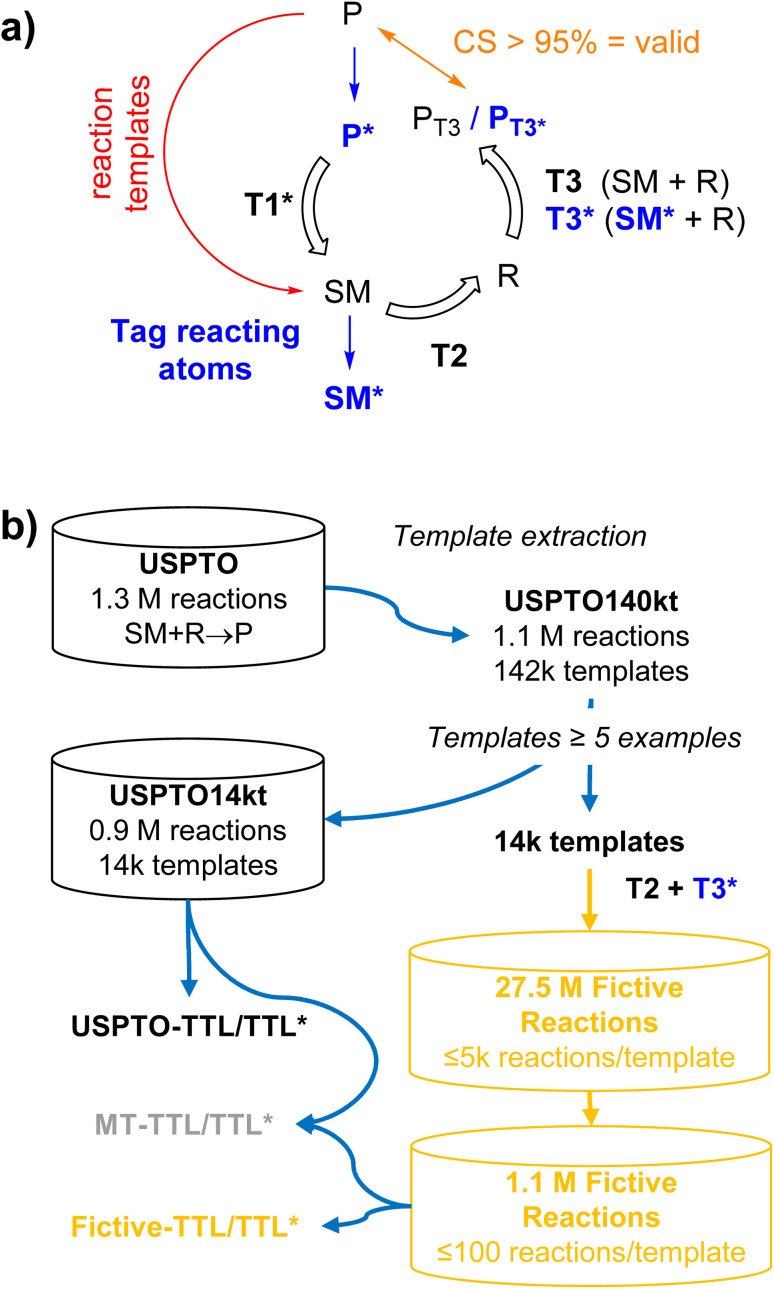
Data augmentation in a triple transformer loop and evaluation for single-step retrosynthesis. (a) Details of the triple transformer loops TTL and TTL*. In the previously described TTL,^27^ the reactive atoms of a product molecule P are first tagged to produce P*. Transformer T1 then predicts the starting materials SM from P*, transformer T2 predicts the reagents R necessary to convert the predicted SM to P, and transformer T3 predicts the product P from the predicted SM + R. In the modified TTL* reported here, the atoms with environmental changes in SM are additionally tagged to produce SM*, and transformer T3* is trained to predict P from SM* + R. In both TTL and TTL*, the reaction SM + R → P or SM* + R → P is validated if the product predicted by T3 or T3* (P_T3_ or P_T3*_) is identical to P with a confidence score >95%. (b) 27.5m fictive reactions were generated by applying 14 024 templates with at least 5 examples, extracted from USPTO, to USPTO molecules as products (P), to generate starting materials (SM), and using transformers T2 to predict reagents (R) and transformer T3* to validate the fictive reactions, up to 5000 reactions per template. For evaluation, TTL models were trained with USPTO or fictive reactions.

Herein we report a new data augmentation approach to enrich datasets in poorly represented reaction types by combining the use of reaction templates with transformer models inspired by our recently reported triple transformer loop (TTL) single-step retrosynthesis model.^27,28^ In the TTL, a product molecule (P) is first tagged at hypothetical atoms with a changing environment to form a series of labeled products (P*), each corresponding to a different bond disconnection. For each P*, a first transformer T1* predicts a starting material (SM), a second transformer T2 predicts reagents (R) from the output of T1*, and a third transformer T3 predicts the product P from the combined outputs of T1* and T2. The reaction is validated if the confidence score (CS) is higher than a chosen threshold, usually CS > 95%. In the data augmentation approach reported here, we do not use T1* but instead generate fictive reactions by applying reaction templates to P to generate a corresponding SM, followed by transformer T2 to predict R. To validate the reaction, we use the atom-mapping information to tag atoms with environmental changes in SM to form a labeled SM*, and request CS > 95%, a value previously found to efficiently select valid reactions,^27,28^ for the prediction of P by a transformer T3* trained to predict P from SM* + R (Fig. 1a).

To obtain a template equilibrated dataset of fictive reactions, we apply our data augmentation approach to 14 024 reaction templates with at least 5 examples in the USPTO dataset to generate up to 5000 reactions per template, resulting in a dataset of 27.5m fictive reactions including reagents (SM + R → P). To test the effect of a template-equilibrated dataset on single-step retrosynthesis, we train a TTL using a subset of this dataset consisting of 1 097 374 fictive reactions containing up to 100 reactions per template, to match the size of the original USPTO dataset, and compare its performance to that of a similar model trained on the USPTO dataset, and to that of a model trained with both datasets simultaneously by multi-task learning, which we have found previously to work well for reaction prediction models (Fig. 1b).^29–31^ Indeed, we find that our template-equilibrated dataset of fictive reactions leads to significant improvements in template-averaged single-step retrosynthesis performance.

## Results and discussion

### Reaction dataset selection and generation of fictive reactions

**Fig. 2:**
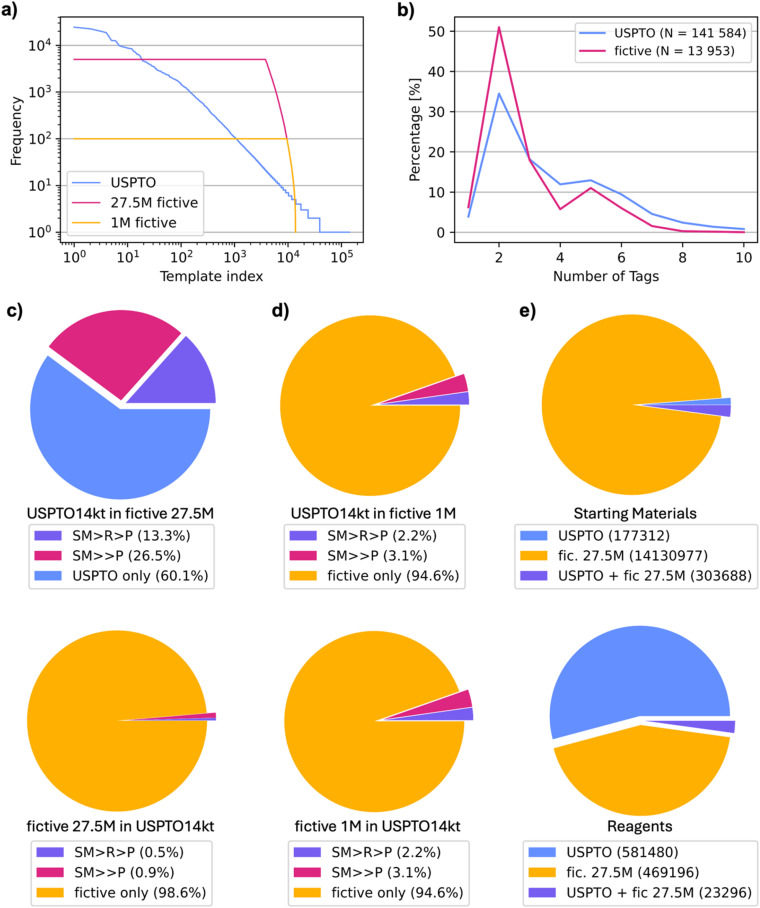
Comparison of fictive and USPTO reactions. (a) 141 584 r1 templates extracted from USPTO and corrected with the templatecorr package, sorted by decreasing number of reactions per template in USPTO140kt (blue line), the 27.5m fictive reactions (red line) and the 1m fictive reaction subset (orange line). (b) Percentage of templates equivalent to a given number of tags in both USPTO140kt (totaling 141 584 unique templates) and the fictive 1m dataset (totaling 13 965 unique templates). (c) Proportions of USPTO14kt reactions shared with the 27.5m fictive reaction dataset and *vice versa*, either exactly shared or under different reaction conditions. (d) Proportions of USPTO14kt reactions shared with the 1m fictive reaction dataset and *vice versa*, either exactly shared or under different reaction conditions. (e) Comparison of the starting materials and reagents present in USPTO14kt and the 27.5m fictive reaction dataset.

We used the United States Patent and Trademark Office (USPTO) chemical reaction dataset,^22,32^ in the modified version listing 1 266 734 USPTO reactions with a single product (P) and between two and ten starting materials (SM).^33^ From this dataset, template extraction for both radius 0 (r0) and radius 1 (r1) templates in SMARTS format was performed using the rxnutils package^34–36^ and succeeded for 1 100 773 reactions, a dataset here named USPTO140kt. Templates were then standardized using the templatecorr package,^37,38^ which resulted in 141 584 unique r1 templates, corresponding to between 1 and 24 523 reactions per template (blue line, Fig. 2a).

We focused the study on the 14 024 r1 templates with at least five example reactions in the USPTO140kt dataset, corresponding to 934 688 reactions (84.9% of the USPTO140kt dataset, hereafter designated as USPTO14kt). These templates corresponded to up to 10 tags, with almost 50% of templates containing two tags. The distribution of these templates to be used for fictive reaction generation was somewhat narrower than in the entire USPTO140kt dataset because many templates with three or more tagged atoms have fewer than five examples (Fig. 2b). For each of the 14 024 reaction templates, we searched the full USPTO dataset for all molecules matching the SMARTS template of the product (P). We then processed each matching molecule by applying the retrosynthesis template to obtain the corresponding starting materials (SM), and transformer T2 of our previously reported TTL^27^ to generate possible reagents R.

Early attempts to validate the resulting fictive reactions SM + R → P by applying transformer T3 of our original TTL to predict P from SM + R with a high confidence score (CS > 95%) resulted in a very low validation rate, which was caused by sensitivity to structural changes in the molecules that were unrelated to the reacting functional groups and often trivial (*e.g.* ethyl *vs.* methyl in a site remote from the reactive site). Fortunately, we found that the validation rate could be increased by identifying atoms with environmental changes in the predicted SM using RXNmapper^39^ to obtain a labeled SM* and using a modified transformer T3* trained with the USPTO140kt reactions to predict P from SM* + R.

We applied the above procedure to each of the 14 024 templates until a maximum of 5000 reactions had been validated for each template. In total, approximately 60 million SM + R precursor pairs were produced by T1 and T2, 38.5 million of which produced the correct P when subjected to T3*. A subset of 27.5 million of these had a confidence score above 95%, covering 13 953 (99.5%) of the 14 024 templates. In this dataset, only 692 templates had fewer reaction examples than in USPTO140kt, while most templates had more reaction examples than in USPTO140kt (red line, Fig. 2a). In view of training a retrosynthesis model, we selected a maximum of 100 reactions per template to form an equilibrated dataset of 1 097 374 validated fictive reactions. In this case, 12 285 of the templates had fewer examples than in USPTO140kt, 1518 templates had more examples than in USPTO140kt, and 150 templates had the same number of examples as in USPTO140kt (orange line, Fig. 2a).

Further comparison of our 27.5m fictive reactions with the USPTO14kt dataset showed that our procedure had regenerated 39.9% of the USPTO14kt dataset when considering SM → P and 13.3% when considering SM + R → P. However, due to their number, most of the 27.5m fictive reactions (98.6% SM → P, 99.5% SM + R → P) were novel compared to USPTO14kt (Fig. 2c). Furthermore, the overlap between USPTO14kt and the 1m subset of our fictive reactions amounted to 6.3% of USPTO14kt (5.4% of the 1m fictive subset) for SM → P reactions and 2.6% of USPTO14kt (2.2% of the 1m fictive subset) for SM + R → P (Fig. 2d). In terms of starting materials, 303 688 of the 481 000 (63.1%) of the SM in USPTO14kt had been regenerated by our fictive reaction generation procedure; however, 14 130 977 (96.7%) of the SM in the 27.5m fictive reaction dataset were novel compared to USPTO14kt. For reagents R, the procedure had generated 469 196 new reagents, while only 23 296 (3.9%) of the 604 776 reagents in USPTO14kt appeared in the fictive reaction dataset, reflecting the selection of templates as well as the effect of transformer T2 in predicting the most probable reagents (Fig. 2e).

**Fig. 3:**
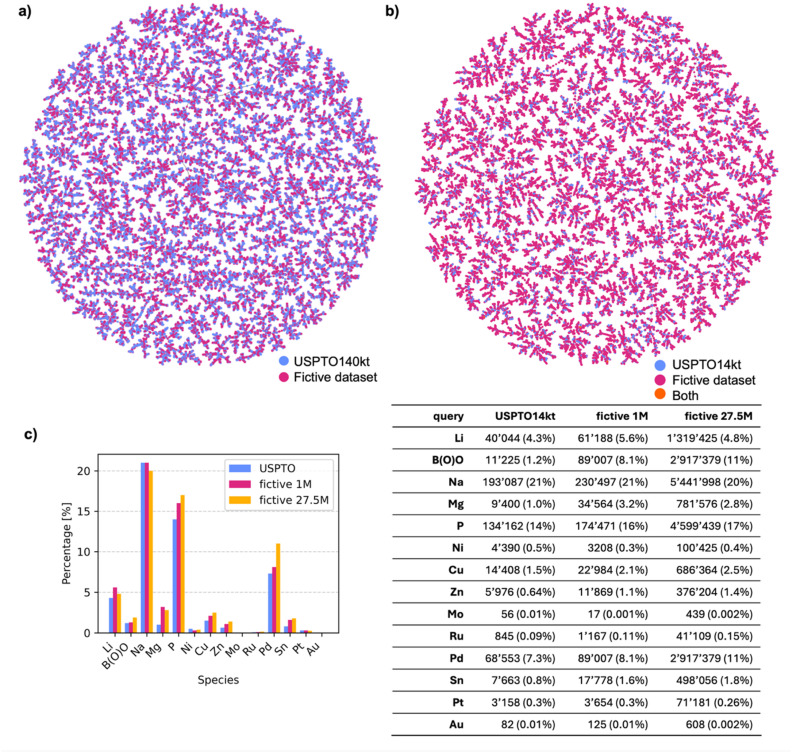
Comparison of USPTO and fictive reactions in terms of chemical space coverage. (a) DRFP TMAP comparing the fictive dataset (∼1m reactions) with USPTO140kt; labels indicate the dataset from which each reaction originates. Each template is represented by 2 randomly picked reactions in each dataset, making a total of 55k reactions. (b) MHFP6 TMAP of starting materials (SM) considering 10 000 SM randomly picked from USPTO14kt and 40 000 SM randomly picked from the 1m fictive reactions. (c) Frequency analysis of reagents by element type in the USPTO14kt, fictive 1m and fictive 27.5m reaction datasets. The presence of such elements/groups highlights characteristic reactivities and catalytic contexts represented in each dataset.

A closer comparison of USPTO and fictive reactions using a TMAP layout,^40^ computed for SM → P reactions using the differential reaction fingerprint DRFP as a similarity measure,^41^ showed that the generated reactions covered a similar chemical space to the original USPTO140kt (Fig. 3a). Similarly, although the vast majority of SM in the fictive reactions were novel compared to USPTO14kt, a TMAP layout using the substructure fingerprint MHFP6 as a similarity measure,^42^ showed that the fictive SM covered the space of USPTO14kt more broadly but in a similar manner (Fig. 3b). Indeed, the overall reaction types remained broadly comparable, as revealed by an analysis of reagents. For instance, sodium (Na) was present in approximately one fifth of the reactions in both USPTO14kt and the fictive datasets, reflecting mostly ester hydrolysis reactions (Fig. 3c). Phosphorus, present mostly in triphenylphosphine (metal-catalyzed processes) and olefination reagents (phosphoranes), increased slightly in the fictive reactions compared to USPTO14kt. Similarly, metals such as magnesium, lithium and zinc used in organometallic processes, as well as other relatively rare elements (Sn, Pt, Ru, and Au), increased significantly in fictive reactions, reflecting the effect of template equilibration.

Taken together, these analyses showed that our data augmentation approach combining templates and TTL transformers allowed us to produce a large, template equilibrated reaction dataset covering a chemical space comparable to the source data.

### Impact of fictive reactions on template-averaged retrosynthesis performance

To assess the practical value of our fictive reaction dataset, we evaluated our approach on the single-step retrosynthesis task in our TTL model. This task is particularly well-suited for testing an equilibrated dataset, as it corrects the overrepresentation of highly frequent transformations while enriching underrepresented reactions, and template-free retrosynthesis models are especially sensitive to such distributional imbalances.^33^ For our comparative evaluation, we trained a reference TTL, here named USPTO-TTL, using the dataset of 934 688 USPTO14kt reactions corresponding to the 14 024 templates, with a train : validation : test set ratio of 80 : 10 : 10, grouping reactions using a common template to avoid data leakage. Using the same procedure, we trained a second TTL, here named fictive-TTL, using the dataset of 1 097 374 fictive reactions, splitting training, validation and test sets with reactions derived from the templates assigned to the corresponding sets in the USPTO-TTL training. Finally, we trained a model using both reaction datasets by multi-task learning, here named MT-TTL. In each case, we also trained models in which the forward validation transformer T3* used starting material SM* with labeled atoms with a change in the environment, labeled USPTO-TTL*, fictive-TTL* and MT-TTL* (Fig. 1b).

To compare the different retrosynthesis models, we measured the single-step round-trip accuracy (RTA), averaged per reaction and starting with the product with tagged atoms. The RTA was introduced by Schwaller *et al.*^43^ and tests the ability of a retrosynthesis model to propose a valid retrosynthetic operation on a product molecule, rather than the ability to reproduce the same retrosynthetic operation as recorded in the test dataset. In addition, we also computed the RTA averaged per template (TA-RTA) to obtain an estimate across different reaction templates, independent of the number of examples per template.

The performances of the different models on the USPTO14kt dataset, which is dominated by a small number of highly populated reaction templates, dropped from approximately 82% on a per reaction basis (RTA) to approximately 65% when averaged per template (TA-RTA) across all three TTLs (Table 1, upper left). For this dataset, the USPTO-TTL performed best in terms of RTA but was overtaken by the fictive-TTL in terms of TA-RTA, while the MT-TTL was in between, reflecting the favorable effect of a template-equilibrated dataset on model performance. A similar effect was visible in the three TTLs*, whose performance was generally higher, taking advantage of starting materials with tagged atoms (dropped from >90% per reaction to ∼80% per template, Table 1, upper right). In this case, however, the fictive-TTL* surpassed the USPTO-TTL* in both RTA and TA-RTA.

**Table 1:** Top 1–3 single step round-trip accuracy (RTA) and template-averaged roundtrip accuracy (TA-RTA) performance for various TTL models on USPTO14kt and fictive dataset test sets

		USPTO-TTL	Fictive-TTL	MT-TTL	USPTO-TTL*	Fictive-TTL*	MT-TTL*
USPTO14kt RTA	Top-1	82.8	78.6	82.7	90.4	**91.9**	91.5
	Top-2	84.5	0.9	84.5	90.8	**92.3**	91.8
	Top-3	85.3	82.0	85.2	90.9	**92.5**	92.0
USPTO14kt TA-RTA	Top-1	62.6	69.0	65.2	78.2	**83.4**	81.7
	Top-2	64.7	71.3	66.9	78.5	**83.8**	82.1
	Top-3	65.8	72.4	67.8	78.7	**84.1**	82.2
Fictive RTA	Top-1	64.3	78.5	70.7	84.1	**92.4**	89.9
	Top-2	66.4	80.5	72.6	85.5	**92.7**	90.1
	Top-3	67.5	81.4	73.5	85.6	**92.9**	90.2
Fictive TA-RTA	Top-1	59.3	73.9	66.0	79.9	**88.4**	85.4
	Top-2	61.4	76.0	67.8	80.2	**88.8**	85.7
	Top-3	62.6	76.8	68.7	80.4	**89.0**	85.8

However, performances on the fictive reaction dataset, which contains the same number of reactions per template, were similar on a per-reaction (RTA) and on a per template (TA-RTA) basis (Table 1, lower half). On this dataset, models trained with USPTO14kt data only (USPTO-TTL and USPTO-TTL*) clearly suffered from the uneven composition of training data with respect to templates, performing ∼60% as TTL and ∼80% as TTL* compared to ∼74% and ∼88% for the corresponding models trained with fictive reactions (fictive-TTL and fictive-TTL*). Again, the MT-TTL and MT-TTL* performed in between the two other models.

**Fig. 4:**
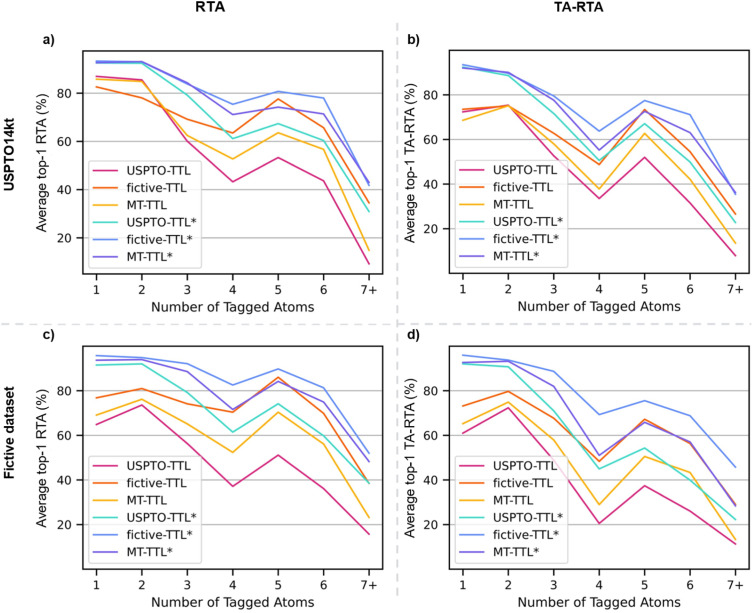
Top-1 average round-trip accuracy per reaction (RTA, (a and c)) and template-averaged round-trip accuracy (TA-RTA, (b and d)) as a function of the number of tagged atoms (*i.e.* the number of atoms undergoing a change in the environment throughout the reaction) for the reaction templates evaluated on USPTO14kt (a and b) and fictive reaction (c and d) test sets. Standard deviations on RTA (20–50%) and TA-RTA (5–44%) are not shown for clarity.

For both the UPSTO and the fictive reactions, performances were highest with the TTLs trained using fictive reactions (fictive-TTL and fictive-TTL*), reflecting the advantage of a template-equilibrated dataset for model training. There was no performance increase with the models trained on both datasets simultaneously (MT-TTL and MT-TTL*). In all cases, using starting materials with tagged atoms provided a strong performance advantage, with the model fictive-TTL* performing best across both test sets on a per reaction and on a per template basis. The same trends appeared when analyzing performance as a function of the number of tagged atoms, serving as an indication of reaction complexity (Fig. 4a and b). For all models, the RTA and TA-RTA dropped at four tagged atoms and strongly decreased for reactions with seven or more tagged atoms. The curves followed the same trend as the number of reactions in the datasets as a function of tagged (reacting) atoms (Fig. 2b). This trend might therefore simply reflect the influence of dataset size, although increasing reaction complexity might also play a role.

## Conclusion

In this work, we presented a new data augmentation approach allowing for the enrichment of all reactivities present in the source dataset. Our approach consisted of generating starting materials (SM) by applying 14 024 r1 reaction templates extracted from the USPTO dataset to USPTO molecules as products (P) and applying transformer models for reagent (R) and product (P) prediction to validate up to 5000 fictive reactions SM + R → P per template. We used the confidence score of transformer models trained on the data as a filter to select meaningful transformations based on established knowledge. By this approach, we obtained a large dataset of 27.5m fictive reactions that covers and expands USPTO14kt's chemical space. A template-equilibrated dataset of 1 097 374 validated fictive reactions containing up to 100 reactions per template was used to evaluate the impact of equilibrated datasets on the single-step retrosynthesis task. We showed that the per-template round-trip accuracy of the non-augmented TTL can be significantly improved by using fictive, template-equilibrated data, and even more so by replacing the forward reaction prediction T3 model with a forward-tag validation model T3*. The fictive reaction dataset presented here could be useful to evaluate different retrosynthesis models, evaluate classification performance or other tasks related to reaction SMILES. Furthermore, the data augmentation scheme can be applied to better exploit the information contained in other open-source datasets.

## Methods

### Datasets and template extraction

We used the United States Patent and Trademark Office (USPTO) chemical reaction dataset,^22,32^ in the modified version by Thakkar *et al.*,^33^ which is limited to reactions with a single product (P) and between 2 and 10 starting materials (SM) and reagents (R). Retrosynthetic reaction templates from this dataset were extracted in SMARTS format with radii 0 and 1 using the rxnutils package.^34–36^ A radius 0 template only carries information concerning atoms whose environment changed throughout the reaction, whereas radius 1 templates also include information for the atoms connected to them. The extraction yielded 95 663 unique template hashes for radius 0 and 262 266 for radius 1. To standardize the syntax of radius 1 templates, we employed the templatecorr package,^37,38^ which requires both radii 0 and 1 templates. The hierarchical correction algorithm uses subgraph isomorphisms on templates sharing the same general template (r0). If several higher radius templates are found to be equivalent, they are all rewritten as the most general and exclusive pattern (we used the published method following the available tutorials as detailed in https://github.com/hesther/templatecorr). The standardization resulted in the dataset here named USPTO140kt featuring 1 100 773 reactions corresponding to 141 584 unique radius 1 templates. Further constraining this dataset to templates with at least five occurrences left the dataset here named USPTO14kt, containing 934 688 reactions from 14 024 radius 1 templates.

### Generating fictive reactions

The generation of fictive reactions started with a pool of 1 505 837 molecules collected from USPTO, split into 1000 subsets. For each of the above-mentioned 14 024 radius 1 templates in SMARTS format, we searched the 1000 subsets of USPTO molecules in random order for molecules matching the product (P) of the template. For each matching molecule, we applied the template to generate the corresponding starting materials (SM) and used transformer T2 of our previously reported TTL^28^ to obtain reagents (R). We then labeled atoms with an environmental change in the template-generated SM using RNXmapper^39^ to obtain SM* with tagged atoms, labeled with a specific token (“!”) placed next to each of the said atoms as described before.^27^ Finally, we predicted P using transformer T3*, trained to predict P from SM* + R with tagged reactant atoms using USPTO140kt (split into 990 391 reactions for training, 55 278 for validation, and 55 104 for testing), and retained the fictive reaction if the confidence score of T3* exceeded 95%. Confidence scores of OpenNMT^44,45^ models have been developed and used in previous work by Kreutter *et al.*^31^

The above procedure was repeated for each of the 14 024 r1 templates until up to 5000 fictive reactions had been validated or all USPTO molecules matching the product side of the template had been tested as products. The procedure succeeded for 13 953 (99.5%) of the 14 024 r1 templates and resulted in 27.5m fictive reactions. A smaller template-equilibrated subset of 1 097 374 fictive reactions was obtained by collecting up to 100 reactions per template.

### Performance evaluation

The possible use of our fictive reactions was evaluated by measuring the single-step retrosynthesis performance of TTL models trained with either USPTO14kt or the similarly sized, smaller subset of fictive reactions mentioned above, or with both sets together in multitask learning using each 0.5 weight coefficient using the OpenNMT library.^44,45^ Datasets were split so that all reactions, from both the USPTO14kt subset and the fictive reaction subset, belonging to the same reaction template would be in the same split (training, validation or test set), for an overall 80 : 10 : 10. TTL transformer models were trained using our previously reported procedures,^27,28^ whereby the validation transformer T3 was trained either with unlabeled reactions (USPTO-TTL, fictive-TTL and MT-TTL) or with reactions featuring labeled reactive atoms in SM* (USPTO-TTL*, fictive-TTL* and MT-TTL*). The six different models were compared with the round-trip accuracy metric (RTA), measuring the frequency with which the product (P) is regenerated by the TTL among the list of top-N predictions, averaged across all reactions (RTA), or averaged per template (TA-RTA).

## Author contributions

YG designed and carried out the study and wrote the paper, DK designed the study and gave technical guidance, and JLR designed and supervised the study and wrote the paper.

## Conflicts of interest

The authors declare that they have no competing interests.

## Data Availability

Code availability: the rxnutils package used for template extraction is available at https://github.com/MolecularAI/reaction_utils. The templatecorr package used to correct r1 templates is available at https://github.com/hesther/templatecorr. The code of the enrichment framework is available at https://github.com/yvsgrndjn/USPTO_balance. The USPTO version from Thakkar *et al.* can be found in their Zenodo repository.^33,46^ The dataset of fictive reactions created in this work (27.5m reactions) is available on Zenodo at https://doi.org/10.5281/zenodo.13120462. The equilibrated fictive dataset of 1 097 374 reactions with up to 100 reactions per reaction template is available at https://doi.org/10.5281/zenodo.17301372. TMAPs from Fig. 3 are available as interactive plots on Zenodo: https://zenodo.org/records/17300855.
